# Synthesis and Preliminary Evaluation of a New ^99m^Tc Labeled Substance P Analogue as a Potential Tumor Imaging Agent

**Published:** 2015

**Authors:** Saeed Mozaffari, Mostafa Erfani, Davood Beiki, Fariba Johari Daha, Farzad Kobarfard, Saeed Balalaie, Babak Fallahi

**Affiliations:** a *Department of Radiopharmacy, School of Pharmacy, Tehran University of Medical Sciences, Tehran, Iran. *; b *Nuclear Science Research School, Nuclear Science and Technology Research Institute (NSTRI), Atomic Energy Organization of Iran (AEOI), Tehran, Iran. *; c *Research Center for Nuclear Medicine, Tehran University of Medical Sciences, Tehran, Iran. *; d *Department of Medicinal Chemistry, School of Pharmacy, Shahid Beheshti University of Medical Sciences, Tehran, Iran. *; e *Peptide Chemistry Research Center, K. N. Toosi University of Technology, Tehran, Iran.*

**Keywords:** NK_1_R, Substance P analogue, ^ 99m^Tc, HYNIC, U373MG cells

## Abstract

Neurokinin 1 receptors (NK_1_R) are overexpressed on several types of important human cancer cells. Substance P (SP) is the most specific endogenous ligand known for NK_1_Rs. Accordingly,a new SP analogue was synthesized and evaluated for detection of NK_1_R positive tumors.[6-hydrazinopyridine-3-carboxylic acid (HYNIC)-Tyr^8^-Met(O)^11^-SP] was synthesized and radiolabeled with ^99m^Tc using ethylenediamine-N,N'-diacetic acid (EDDA)and Tricine as coligands. Common physicochemical properties of radioconjugate were studied and *in-vitro* cell line biological tests were accomplished to determine the receptor mediated characteristics. *In-vivo *biodistribution in normal and tumor bearingnude mice was also assessed. The cold peptide was prepared in high purity (>99%) and radiolabeled with ^99m^Tc at high specific activities (84-112GBq/µmol) with an acceptable labeling yield (>95%). The radioconjugate was stable *in-vitro* in the presence of human serum and showed 44% protein binding to human serumalbumin. *In-vitro* cell line studies on U373MG cells showed an acceptable uptake up to 4.91 ± 0.22% with the ratio of 60.21 ± 1.19% for its specific fraction and increasing specific internalization during 4 h. Receptor binding assays on U373MG cells indicated a mean Kd of 2.46 ± 0.43 nM and Bmax of 128925 ± 8145 sites/cell. *In-vivo* investigations determined the specific tumor uptake in 3.36 percent of injected dose per gram (%ID/g) for U373MG cells and noticeable accumulations of activity in the intestines and lung. Predominant renal excretion pathway was demonstrated.

Therefore, this new radiolabeled peptide could be a promising radiotracer for detection of NK_1_R positive primary or secondary tumors.

## Introduction

Radiolabeled peptides have shown notable applications fordiagnosis and therapy of tumorsin the latest decades(1-4) and play an important role in receptor targeting of tumors (5-7).Up to now, several types of peptides have been introduced for tumor targeting and many types of cancer cells were demonstrated overexpression of various peptide receptors (8-10).

Substance P (SP) is an undecapeptide which belongs to a family of neuropeptides known as tachykinins with the common C-terminal sequence Phe-X-Gly-Leu-Met-NH_2_. Until now three different transmembrane G-protein coupled receptors named Neurokinin 1- 3 receptors (NK_1_R, NK_2_R and NK_3_R) are known for biological functions of tachykinins. The number ordering in NKRs nomenclature is according to the preferred affinity of them to the SP, NKA, and NKB, respectively. Thus, SP is the most specific endogenous ligand known for NK_1_Rs(11, 12). SP is the mediator of many biological actions both in central nervous system (CNS) and peripheral nervous system (PNS) independently in µM and nM concentrations. The physiological presence of NK_1_Rs have been reported in several types of normal tissues including brain, salivary glands, thymus, lymphatic tissues, smooth muscles of the gastrointestinal and respiratory tracts. SP plays its physiological roles as a vasoactive, secretary, and mitogenic factor (13-15). This neuropeptide is also an important mediator of some pathological conditions such as emesis, inflammation, wound healing, pain, anxiety and depression (15-17). Additionally, SP has been recognized as a stimulator of angiogenesis and mitosis in the progression of various malignancies with increased expression of NK_1_Rsdescribed in several tumors including glioma (GBM), colorectal carcinoma, pancreatic carcinoma, breast carcinoma, retinoblastoma, neuroblastoma, melanoma, laryngeal carcinoma, and oral squamous cell carcinoma (18-21). Since SP could be developing compound in several cancer cell types via NK_1_Rs, numerous approaches to design a specific radiopharmaceuticals which target the NK_1_Rs have been described applying SP analogues and other compounds (22-26). Nevertheless, more efforts are needed for development of these agents. With this concept, the aim of the present study was synthesis and ^99m^Tc radiolabeling of a new SP analogue, and to evaluate potential affinity of the mentioned radiolabeled peptide by *in-vitro* and *in-vivo* tests on human malignant astrocytoma cell line (U373MG) as a model for NK_1_R expressing tumors.

## Experimental

*General*

Rink amide MBHA (4-methylbenzhydrylamine) resin and all of the Fmoc-protected amino acids were purchased from Nova Biochem. Boc-HYNIC reagent was synthesized as previously reported method by Abrams *et al. *(27). Solvents, coupling reagents, pyridine, and other reagents were purchased from Fluka, and used without further purification. All aqueous solutions were made using double distilled ultrapure water and filtered by 22 µ filters before biological uses. The cell lines were cultured in Dulbecco’s Modified Eagle’s Medium (DMEM) and Roswell Park Memorial Institute (RPMI 1640) mediums supplemented with 10% (v/v) fetal bovine serum (FBS), 2mM L-Gln, 100 units/mL penicillinG, 100 µg/mL streptomycin from Gibco at 37 °C, 95% humidity, 5% CO_2_ in a sterile incubator. Sodium pertechnetate (Na^99m^TcO_4_) was obtained from commercially available^99^Mo/^99m^Tc generator (AEOI, Tehran, Iran).A JASCO 880-PU intelligent pump reverse phase high performance liquid chromatography (RP-HPLC) with a multiwave length UV detector and a flow-through Raytest-Gabi γ-detector was used for HPLC analysis. A CC 250×4.6mmNucleosil 120-5 C18 column from Teknokroma was used for analytical HPLC, and a VP 250×30 mm Nucleosil 100-5 C18 column was used for semi-preparative HPLC purification. The mobile phase was 0.1% (v/v)TFA/H_2_O (Solvent A) and ACN (Solvent B)with the following gradients. Method I for analytical HPLC: 0-5 min 5% B (95% A), 5-20 min 5-100% B, 20-25 min 100% B, 25-30 min 100-5% B, 30-35 min 5% B; flow = 1 mL/min; λ = 280 nm. Method II for semi-preparative HPLC: 0-2 min 20% B (80% A), 2-17 min 20-50% B, 17-19 min 50-100% B), 19-21 min 100% B), 21-25 min 100-20% B, 25-27 min 20% B; flow = 15 ml/min, λ = 280 nm. Mass spectrum was recorded on an Agilent 1100/Bruker Daltonic (Ion trap) VL instrument (LC-MS) using electro-sprayionization (ESI) mode. Quantitative gamma counting was performed on a well-type NaI γ-counter EG&G/ORTEC Model 4001M. Male albino mice and male athymic nude mice were obtained from animal house of research laboratories (AEOI, Tehran, Iran). U373MG cells were obtained from National Cell bank of Iran (NCBI, Pasteur Institute of Iran).

*Synthesis*

HYNIC-Tyr^8^-Met(O)^11^-SP was synthesized according to the standard Fmoc solid phase peptide synthesis chemistry on Rink Amide MBHAresin with loading capacity of 0.69 mmol/g. Briefly, the treatment of diisopropylcarbodiimide (DIC) and N-hydroxybenzotriazole (HOBT)activated carboxyl groups of the Fmoc amino acids to react with the N-terminal amino groups of growing peptide on Rink Amide MBHA resinfor stepwise amino acid addition. The carbodiimide/HOBT coupling strategy was used to minimize the racemization of chiral amino acids and to increase the yield of reactions as the strategy needs equimolar application of amino acids and coupling reagents theoretically. Coupling of each amino acid was performed in the presence of 3 mol excess of Fmocamino acid, 3 mol excess of HOBT, 3 mol excess of DIC and 5 mol excess of diisopropylethylamine (DIPEA) in dimethylformamide (DMF), even though the first Fmoc amino acid was coupled to amino groups of the resin in the presence of 5 mol excess of reagents to achieve the maximum loading on resin active sites. The Kaiser test was used to assess the fullness of coupling reactions and the Fmoc groups were removed by adding 20% piperidine in DMF. Finally, coupling of Boc-HYNIC to peptide was performed in the presence of 1.2 mol excess of Boc-HYNIC, 2.5 mol excess of 2-(7-aza-1H-benzotriazole-1-yl)-1,1,3,3-tetramethyluronium hexafluorophosphate (HATU), and 5 mol excess of DIPEA in DMF. The cleavage step from the resin and the final deprotection of all remained protecting groups was done in a standard cocktail containing trifluoroaceticacid (TFA), triisopropylsilane (TIPS), thioanisole, and water (92.5:2.5:2.5:2.5).The crude peptide was precipitated into cold petroleum ether/diisopropyl ether (50:50). Then, the precipitate was dissolved in water/methanol (50:50) and purified by semi-preparative RP-HPLC (method II).The purified product was lyophilized in vacuum and characterized by analytical HPLC and LC-MS.

*Radiolabeling with* ^99m^ *Tc*

Labeling of HYNIC-peptide was performed as previously described (28-31) with ^99m^Tc in the presence of ethylenediamine-N,N'-diacetic acid (EDDA) and tris(hydroxymethyl)methylglycine (Tricine) as coligands using an exchange labeling approach with both present together. In brief, the labeling process was done by adding 10.75 μL of the stock solution (20 μg of peptide), 15 mg of Tricine and 5 mg of EDDA as coligands in 0.5 mL of water. Then, 40 μg SnCl_2_ (10 μL of freshly prepared 4 mg/mL SnCl_2_in nitrogen-purged 0.1 M HCl) was added to this solution. Instantly, 30-35 mCi (1110-1295MBq) of freshly eluted sodium pertechnetate (Na^99m^TcO_4_) in 0.5 mL normal saline was added to the solution and incubated for 15 min at 95 °C in a shielded container. After cooling down to room temperature, the labeling yield was checked on RP-HPLC.

*In-vitro evaluation of radiolabeled peptides*

*Stability in aqueous solution and human serum*

The stability of radiolabeled peptide in aqueous solution was evaluated by incubation of the reaction mixture at room temperature (25 °C)up to 24 h.Stability in human serum at 37 °C was tested in parallel after adding 100 μL of reaction mixture to 1ml of fresh human serum. The incubation mixtures were sampled at 1, 4, and 24 h time points. Serum samples (100-200 μL) were treated with ethanol (200-400 μL) and centrifuged (4000 g, 5 min, 4 °C) to precipitate the serum proteins. 20-100 μL aliquots from the supernatant were separated to assess the degradation of ^99m^Tc labeled peptide by RP-HPLC (method I). 100 μL aliquots from the 25 °C incubated mixture were passed through a 22 μ filter to eliminate possible ^99m^Tc colloids and then, 5-20 μL of filtrate were analyzed by RP-HPLC as well.

*Protein binding*

For protein binding estimation, freshly labeled peptide solution was filtered through a 22 μ filter and a 100 μL aliquot of filtrate was incubated in 1ml fresh human serum for 2 h. After ethanol treatment and centrifugation (4000 g, 5 min, 4 °C), all the supernatant was completely removed. A slight wash with 1x phosphate-buffered saline (PBS) was done on the surface of the sediment and the eluate was added to the removed supernatant. The total activity of the supernatant and the activity of the sediment were measured by well-type NaI γ-counter to determine the percentage of radio peptide bound or transferred to albumin and other serum proteins.

*Log p-values*

In a 2 mL microtube, 0.5 mL of the^99m^Tc labeled peptides in PBS was mixed with 0.5 mL of octanol. The tube was vigorously vortexed over a period of 10 min and centrifuged at 4000g for 5 min. Three aliquots of 100 μL were sampled from eachlayer and counted in theγ counter. The averaged activities from the aqueous and the octanol layers were used to calculate the log p-values. The octanol-to-water partition coefficient (P_o/w_) of the radiolabeled peptides was calculated by dividing the counts of the octanol phase by that of the aqueous phase.

*In-vitro cell line studies*

All the main cell studies were performed on U373MG human Glioblastoma cell lines. Cells were cultured in DMEM was supplemented with L-glutamine, antibiotics (penicillin, streptomycine, gentamycine) and10% FBS. They were grown in culture until a sufficient number of cells were available. At proper times, cells were detached by trypsin-EDTA (0.25% and 0.02%) from the culture flask and diluted with fresh medium to start a new culture or the cell line tests.

*Internalization and surface binding studies in U373MG cell lines*

Internalization of the ^99m^Tclabeled HYNIC-Tyr^8^-Met(O)^11^-SP was studied as previously described method(32, 33) with some modifications. In brief, newly detached U373MG cells were seeded in six-well plates 12 h before the experiment. Their culture medium was replaced by 1.2 mL of previously warmed fresh medium and cells were allowed to adjust to the medium for 1 h at 37 °C, 5% CO_2_. In order to determine nonspecific internalization, one line of wells per plate was incubated with 10000 fold excess of unlabeled SP (25 nmol peptide in 150 μL PBS per well) at 37°C/5% CO_2_ for 15 min to block NK_1_Rs. Then about 150kBq (2.5 pmol peptide) in 150 μL diluted solution of freshly radiolabeled peptide was added to each of the wells and were incubated (in triplicates) for 0.5, 1, 2 and 4 h at 37 °C/5% CO_2_, respectively. As the final volume of blocked wells was 1.5 mL, the content volume of unblocked wells was adjusted to 1.5 mL by adding 150 μL PBS per well. At each time point, radioactive media were aspirated and wells were washed twice with cold PBS (1x, pH 7.2) to remove free radio peptides. After that, cells were treated with 1 mLGly buffer (0.2M, pH adjusted to 2.8 with 1 M HCl) at room temperature for 5 min (twice) to remove cell-surface bound radio peptide. Finally, the cells were detached from the plates by three steps incubation with 1N NaOH at room temperature for 5 min to determine the internalized radio peptide. All consequent fractions were measured with a NaI γ-counter and results were expressed as percentage of total activity per well (free + cell-surface bound + internalized). Nonspecific accumulations were subtracted from totals to determine the specific accumulations.

*Saturation binding experiment (Kd and Bmax)*

For saturation binding experiment, U373MG cells were incubated with increasing concentrations of ^99m^Tc labeled HYNIC-Tyr^8^-Met(O)^11^-SP in the presence and absence of excess unlabeled SP to determine nonspecific (NSB) and total binding (T), respectively. In brief, newly detached U373MG cells were suspended in fresh RPMI medium and aliquots of 800000 cell/1mL were prepared in 5 mL test tubes. In a series of tubes, various concentrations of radiolabeled peptide (0.01-10 nM) in triplicate were the only added but in the other series the radiolabeled peptide was added as well with a fixed amount of excess unlabeled SP. The tubes were incubated for 90 min with a slight shaking every 5 min and were centrifuged at 200 g. Then the supernatants were removed and the surface of cell pellets were washed with cold PBS 1x slightly. The radioactivity of the supernatants was counted to determine the free radiopeptide amounts. The radioactivity of the pellets was counted to measure the NSB and T. The affinity for the radio ligand (Kd) and the maximal number of receptor binding sites (Bmax) were calculated from specific binding (SB) curve using the nonlinear regression analysis of Graphpad Prism 5^th^ (SB = T - NSB).

*In-vivo evaluation of radiolabeled peptide*

Male albino mice in a weight range of18-24 g were used for normal mice treatments and malenude mice (6 to 8 weeks old) were used for *in-vivo* tumor treatments. The mice were housed at controlled room temperature (25 ºC) in a 12 h light/12 h dark schedule. They were kept in standard cages with free access to food and water except during the experiments. The nude mice were grown and kept in isolated sterile condition. All animal experiments were carried out in compliance with our institutional ethical guidelines.

*Biodistribution in normal mice*

Freshly labeled ^99m^Tc-HYNIC-Tyr^8^-Met(O)^11^-SP (purity>98%) was diluted to 3.3 nmol/mL with normal saline and a dose of 800 µCi/ 330 pmol in 100 µL was injected to each mouse via the tail vein. Also agroup of three animals (blocked group) were given150 µg of SP in 50 µL salineco-injected with the radiopeptide to determine the non-specific biodistribution of the radiopeptide by blocking the receptor-positive organs. After 1, 4, and 24 h post injection the mice in groups of three animals were sacrificed and organs of interest were excised. All organs and tissues were weighed, and the radioactivity was determined by gamma counter. Results were expressed as percentage of injected dose per gram (% ID/g) of tissue.

*Biodistribution in tumor bearing nude mice*

U373MG cells were grown and harvested by trypsinization. Cells were washed twice with fresh RPMI medium containing 20% FBS and centrifuged for 5 min at 200 g. Pellets were resuspended in RPMI medium containing 20% FBS. A 100 µL suspension of 4 × 10^6^ cells was subcutaneously injected on the right flank of each nude mouse. After four weeks, the size of inducted tumors was suitable for biodistribution study and mice were injected via the tail vein with radiolabeled peptide in two groups in the presence and absence of block. They sacrificed at 1 h and 4 h time points to assay the biodistribution. They were dissected and % ID/g of tissues was measured as described above. Finally, tumor-to-organ ratios were calculated.

*Statistical analysis*

Means and standard deviations for internalization study were computed on Microsoft Excel. Student’s t-test by GraphPad Prism was used to determine statistical significances for internalization and biodistribution study. Associated analyzing method by GraphPad Prism was accomplished to calculate the Kd and Bmax. The 95% level was considered for confidence intervals and significant differences as the default setting.

## Results and Discussion

*Synthesis, labeling and in-vitro evaluation*

**Figure 1:**
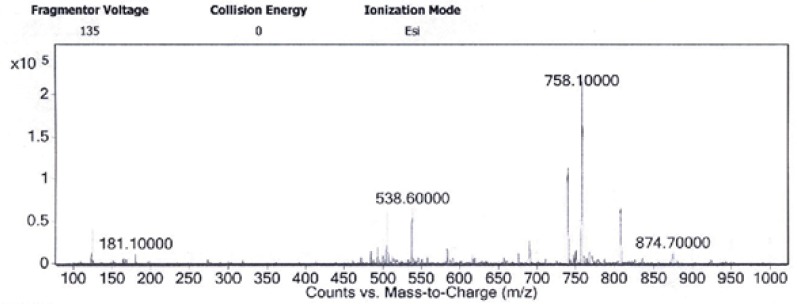
ESI-Mass spectra of HYNIC-Tyr^8^-Met(O)^11^-SP obtained by LC-MS analysis. Isolated [M+2H]^2+^ molecular ion at 758.1 m/z confirms the 1514.7 molecular mass of the HYNIC-Tyr^8^-Met(O)^11^-SP

This newHYNIC-Tyr^8^-Met(O)^11^-SPderivative was conveniently synthesized by solid phase peptide synthesis method on Rink Amide MBHA resin via Fmoc strategy followed by Boc strategy for N-terminal conjugation of Boc-HYNIC which was deprotected during the cleavage step. Yields of all amino acid coupling steps were in a range of 95-99% and the overall yield of the crude peptide was about 76%. The crude peptide was purified (purity>99%) by semi-preparative RP-HPLC and verified by analytical RP-HPLC and LC-MS (Table 1 and Figure 1). Final yield after purification and lyophilization was approximately 53%. ESI-Mass analysis was consistent with the calculated molecular weight for the HYNIC-Tyr^8^-Met(O)^11^-SP. Calculated mass for this new derivative is 1514.76 g/mol and LC-MS analysis confirmed a [M+2H]^2+^molecular ion at 758.1 m/z.

**Table 1:** Analytical data of HYNIC-Tyr^8^-Met(O)^11^-SP.

**Compound**	**Chemical formula**	**Calculated** **mass (g/mol)**	**Observed** **mass (m/z)**	**RP-HPLC** **Rt (min)**	**Purity (%)**
HYNIC-Tyr^8^-Met(O)^11^-SP	C_69_H_103_N_21_O_16_S	1514.75	758.1 [M+2H]^2+^	12.66	> 99

**Figure 2:**
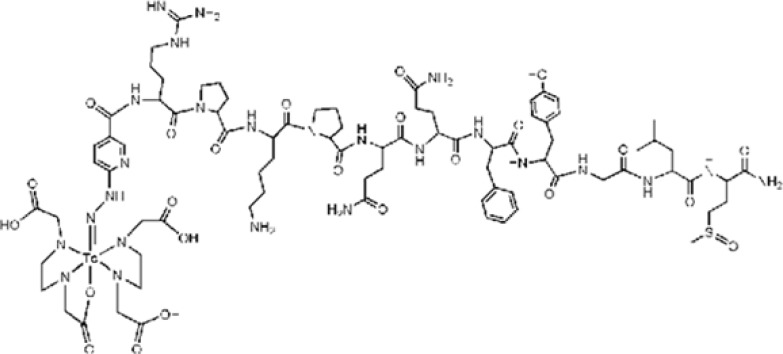
The proposed structure of the prepared EDDA-^99m^Tc-HYNIC^0^-Tyr^8^-Met(O)^11^-SP complex.

**Figure 3:**
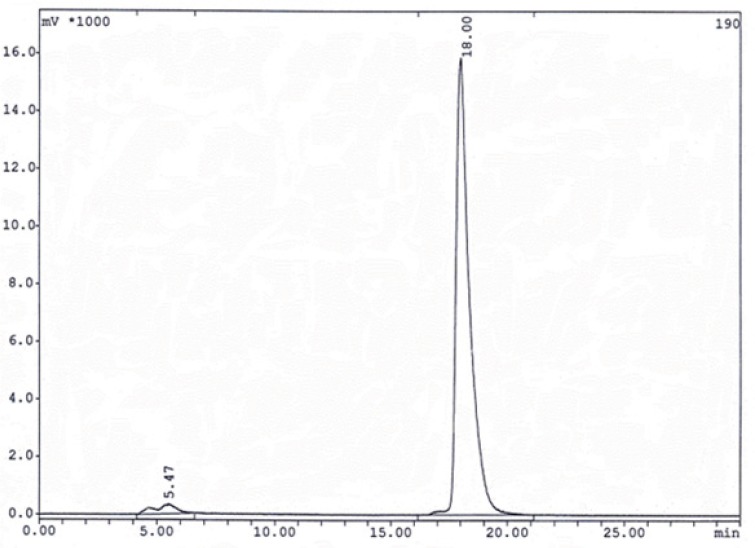
The HPLC γ-radiochromatogram of ^99m^Tc-peptide complexe gradient method I. The retention times are 5.47 min for ^99m^TcO_4_ˉ and 18.00 min for EDDA-^99m^Tc- HYNIC-Tyr^8^-Met(O)^11^-SP.

HYNIC-Tyr^8^-Met(O)^11^-SP was labeled with ^99m^Tc in a repeatable method using EDDA-Tricine labeling approach. The proposed structure of desired complex is shown in Figure 2. Labeling experiments were achieved a high specific activity (30-40 mCi/20µg or 84-112GBq/µmol) resulting acceptable labeling yield (>95%) with a single species at 18 min retention time for EDDA-^99m^Tc-HYNIC-Tyr^8^-Met(O)^11^-SPon RP-HPLC analysis (Figure 3). Stability studies in aqueous solution and human serum confirmed a good stability of radiolabeled complexes with no considerable release of ^99m^TcO_4_ˉor peptide degradation during the observation time.

Protein binding of radiolabeled peptide was measured by centrifugation methodresulting44% protein binding for radio conjugate after 2 h incubation. A value of -3.75 log P was calculated for radiolabeled complex.

*In-vitro and in-vivo cell line evaluations*

**Figure 4:**
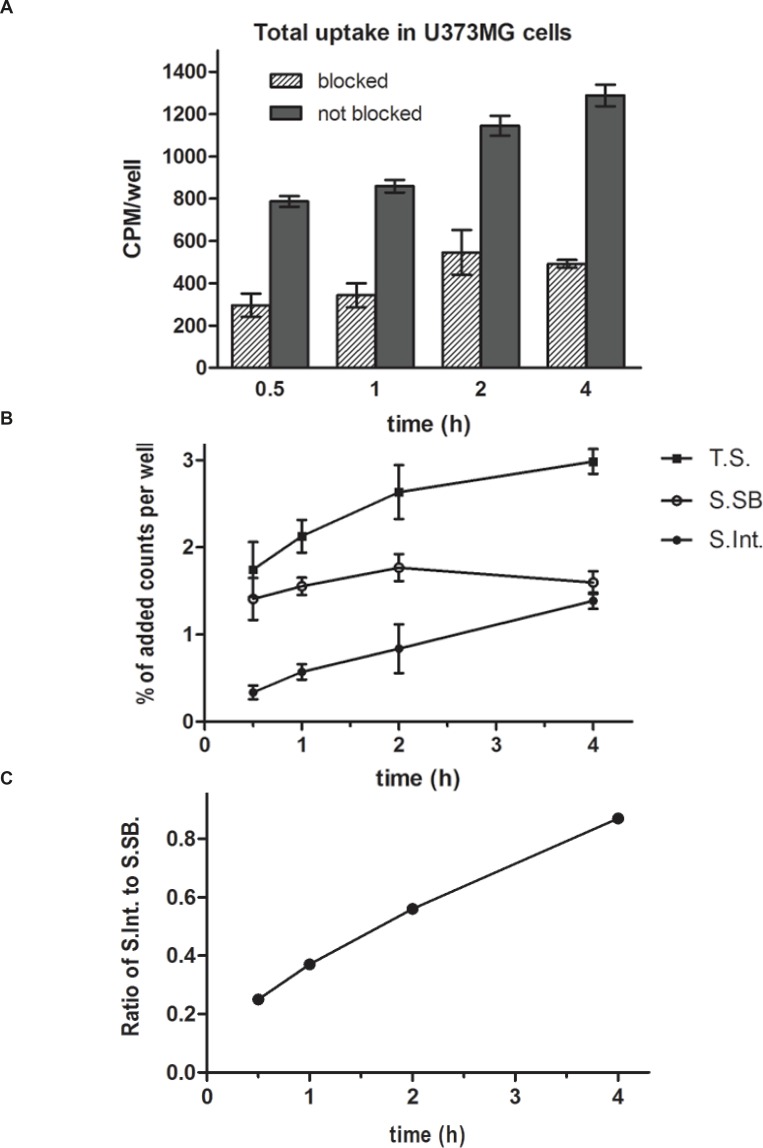
Total counts per minute per wells (CPM/well) at observation time points which shows the uptake of EDDA-^99m^Tc-HYNIC^0^-Tyr^8^-Met(O)^11^-SP into the U373MG cells. The differences between the blocked and not blocked series were significant in all the time points (P<0.01). Values are mean ± SD (4a). Separated percentages of added counts per wells during the time which absorbed to the U373MG cells in two parts, the surface bound fraction of radiopeptides and the internalized portion (4b). The ratio of specific internalized radiopeptides to specific surface bound radiopeptides at 0.5, 1, 2, and 4 h time points shows increased values during the 4 h incubation (4c).

U373MG cells are a good model for detection of NK_1_Rs as they endogenously express the full-length NK_1_R (34). Internalization study of the radiolabeled peptide on U373MG cells showed a rapid binding to the cell membrane after 0.5 h and a slight increase in specific internalization during 4 h. Total cell uptake was 2.86 ± 0.10 % at 0.5 h which increased to 4.91 ± 0.22 % up to 4 hand receptor specific cell uptake was 1.71 % at 0.5 h which increased to 2.98 % up to 4 h. It is noticeable that the ratio of specific uptake to total uptake was in a partly constant range (60.21 ± 1.19%). Interestingly, the specific surface-bound activity(acid-removable fraction) reached to a steady state (gentle slope) after 1hwhile the ratio of specific internalized activity (acid-resistant fraction)to specific surface-bound activity was increased during the experiment time (Figure 4a, 4b). This fact means that the binding of ^99m^Tc labeled HYNIC-Tyr^8^-Met(O)^11^-SP to the surface of U373MG cells could be saturable.

Values are calculated from the means of three independent experiments.

Total specific uptake (T.S) = Specific surface bound (S.SB) + Specific internalized (S.Int.)

**Figure 5:**
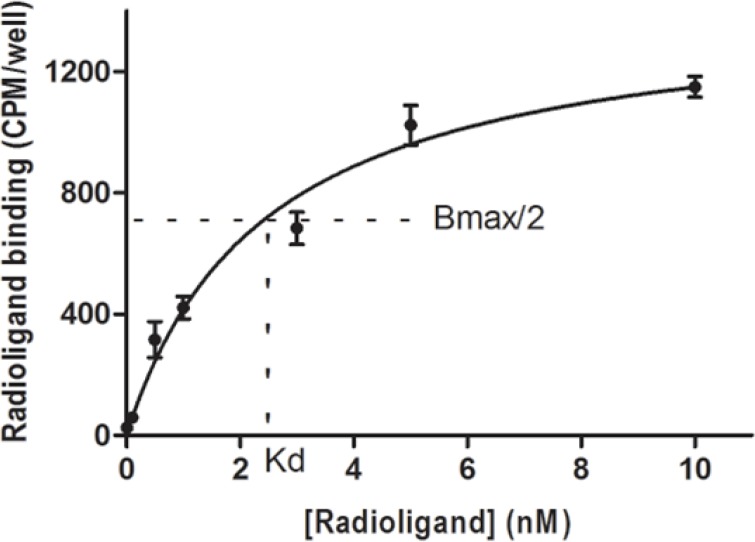
Saturation curve for binding of EDDA- ^99m^Tc-HYNIC^0^-Tyr^8^-Met(O)^11^-SP to U373MG cells which concluded a mean Kd of 2.46 ± 0.43 nM and a mean Bmax of 128925 ± 8145 sites/cell.

Saturation binding experiment on U373MG cells concluded a mean Kd of 2.46 ± 0.43 nM and a mean Bmax of 128925 ± 8145 sites/cell. Related saturation curve can be seen in Figure 5.

**Figure 6:**
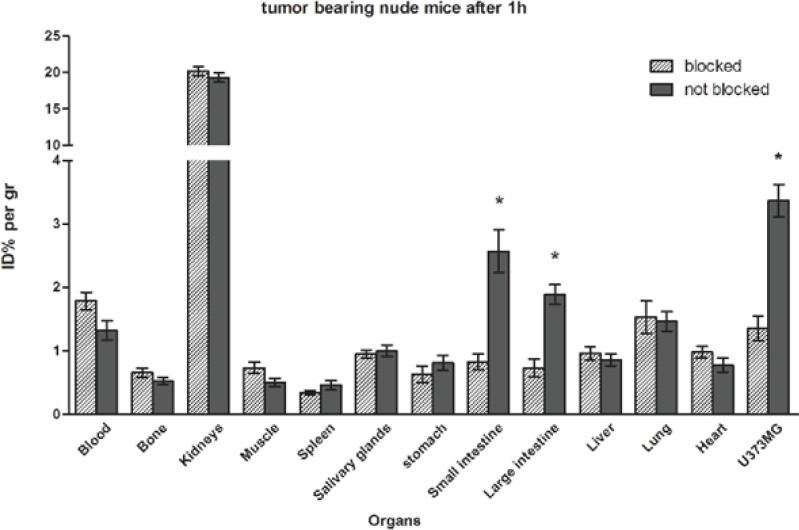
Biodistribution of EDDA- ^99m^Tc-HYNIC^0^-Tyr^8^-Met(O)^11^-SP in tumor bearing nude mice. Data are mean ± SEM of % ID/g obtained from three animals per group

Results from biodistribution evaluation of EDDA-^99m^Tc-HYNIC-Tyr^8^-Met(O)^11^-SPare presented in Table 2 and Figure 6. Both in normal mice and in tumor bearing nude mice, the highest uptake was observed in kidneys (kidneys %ID/g ≥ 20 at 1 h and %ID/g ≥ 13 after 4 h) and generally, a rapid elimination via the renal pathway could be observed. Uptake values in liver and heart were lower than 0.6 %ID/g that confirmed the hydrophilicity of the radiolabeled peptide. 1.02 %ID/g at 1h and 0.36 %ID/g at 4 h about blood, indicates moderate clearance from the circulation. Decreased percent (%D) in uptakes of tissues during the time was lower than 5% for large intestine and in a range of 30-40% for kidneys, liver, and spleen while it was higher than about 50% for other organs from 1 h to 4 h.

**Table 2:** Biodistribution of ^99m^Tc-HYNIC-Tyr^8^-Met(O)^11^-SP in mice

**Organ**	**1 h** **(normal)**	**1 h** **(nude)**	**1h** **(blocked)**	**4 h** **(normal)**	**4 h** **(nude)**	**4 h** **(blocked)**	**24 h** **(normal)**	**D% ** ^a^ **(normal)**	**D% ** ^a^ **(nude)**
Blood	1.02±0.16	1.32±0.16	1.70±0.14	0.36±0.03	0.41±0.12	0.52±0.15	0.14±0.02	65	69
Bone	0.44±0.03	0.52±0.06	0.66±0.07	0.20±0.02	0.27±0.03	0.31±0.03	0.07±0.03	55	48
Kidneys	21.20±0.70	19.3±0.65	20.2±0.61	13.80±1.96	12.76±0.52	13.06±0.76	5.47±0.56	35	34
Muscle	0.32±0.07	0.51±0.06	0.73±0.09	0.09±0.03	0.08±0.01	0.09±0.01	0.04±0.02	72	84
Spleen	0.40±0.09	0.46±0.07	0.34±0.04	0.28±0.06	0.32±0.03	0.26±0.03	0.13±0.08	30	39
Salivary G.	0.61±0.05	1.00±0.09	0.95±0.06	0.26±0.02	0.33±0.04	0.41±0.03	0.20±0.02	57	66
Stomach	1.57±0.13	0.81±0.12	0.63±0.13	0.39±0.06	0.32±0.03	0.26±0.05	0.15±0.01	75	61
S. intestine	2.13±0.17	2.57±0.34	0.83±0.12**	0.89±0.21	0.78±0.07	0.36±0.09*	0.24±0.04	58	70
L. intestine	1.14±0.09	1.89±0.15	0.73±0.14**	1.33±0.28	1.83±0.06	0.70±0.16**	0.37±0.06	-17	3
Liver	0.58±0.04	0.86±0.09	0.96±0.10	0.38±0.05	0.54±0.07	0.50±0.08	0.16±0.02	34	37
Lung	0.94±0.19	1.47±0.15	1.53±0.25	0.33±0.02	0.76±0.12	0.67±0.09	0.17±0.04	65	48
Heart	0.55±0.14	0.78±0.11	0.98±0.09	0.17±0.01	0.25±0.02	0.23±0.02	0.06±0.04	68	68
Tumor	**--------------**	3.36±0.26	1.36±0.19**	**--------------**	1.26±0.11	0.54±0.08**	**--------------**	**----**	62

The data of blocked columns are from blocked tumor bearing nude mice. Values are expressed as %ID/g Mean ± SEM.

a=D% stands for Decreased percent of %ID/g between 1 h and 4 h which is calculated by the formula [(1-4)h D% **=** 100 × (% ID/g^ 1h^ - % ID/g^ 4h^)**/** % ID/g^ 1h^].

* P< 0.05 and

** P< 0.01 when comparing blocked with nonblocked nude mice.

Except the kidneys, uptake in the stomach, intestines, and lung was more than other organs, %ID/g ≥ 1 at 1 hand %ID/g ≥ 0.34 at 4 h vs. %ID/g ≤ 0.6 at 1 h and %ID/g ≤ 0.38 at 4 h for other organs. So, they could be the main target organs of EDDA-^99m^Tc-HYNIC-Tyr^8^-Met(O)^11^-SP. The salivary glands are the other target organs as their uptake is partly high in comparison to other organs like bone and muscle, 0.61 %ID/g vs. 0.44 and 0.32 %ID/g at 1h, respectively. Data from tumor bearing nude mice shows a high uptake value in the U373MG tumors, beside the moderate retention in the circulation after 1 h, 3.36 %ID/g for U373MG tumor vs. 1.32%ID/g for blood. Also, the 7.02 ratio of tumor to muscle (T/M) at 1 h after injection was calculated. In animals co-injected with excess cold SP, the uptake in U373MG tumor and intestines was significantly blocked (P<0.05).

In this study, we report the synthesis and radiolabeling of HYNIC-Tyr^8^-Met(O)^11^-SP for *in-vivo* imaging of NK_1_R positive tumor and their metastases. Some other studies have been previously published applying direct labeling methods or using the BFCA with different radioisotopes(23-25, 35). The use of direct radiometal labeling methods on peptides is to some extent problematic including unpredictable complex formation site which may decrease the receptor affinity of the peptide, increasing the proteolytic degradation, unfavorable biodistribution and extremely rapid blood clearance. These problems could be diminished by linking a proper BFCA on a suitable site on the peptides. Also, SP has been labeled with ^18^F in the form of HYNIC-SP, but a less convenient method for synthesis of cold HYNIC-SP has achieved due to the liquid phase strategy at final coupling step beside the low reactivity of Boc-HYNIC succinimidyl ester and there was not a biological study on NKRs(36). According to the literature, SP is a potent neuropeptide with a high affinity (0.1 to 1nM) for NK_1_Rs (22, 37, 38). The C-terminal portion of the SP, especially amino acids 6 to 11, is the most important part of its sequence for specific interaction with NK_1_R(39-42). For example, the two phenyl alanines at positions 7 and 8 have the most determinant effect for specific binding to NK_1_R. The substitution of these amino acids with nonaromatic amino acids like Val, especially at position 8, changes its affinity from NK_1_R to other NKRs (NK_2_R and NK_3_R). Met C-terminal amide forms a number of important contacts with the NK_1_R backbone (42). The thioether side-chain of Met is susceptible to oxidation reaction during the synthesis and labeling procedures. Complete oxidation of Met to its sulphone [Met(O)_2_] takes place only under severe conditions, and could not be a problem during the synthesis procedure. However, partial oxidation to methionine sulphoxide [Met(O)] can occur upon prolonged exposure to air and other active reagents. Partial oxidation of the thioether may complicate handling and analysis of the peptide after the synthesis step; including the interpretation of analytical HPLC chromatograms, radiolabeling step and other *in-vitro* and *in-vivo* tests(25, 43-46). Any substitution of D-amino acids in sequences makes antagonistic effects on its receptors(47-49).Consequently, the [Met(O)] containing sequence was synthesized withslight modification at position 8 to keep affinity on NK_1_R beside the prevention of further complex impurities from Met oxidation and obtain the lesser complicated results. Phe 8 was replaced with Tyr as another aromatic amino acid which has the chance to make any available H-binding to the receptor site by its –OH group. HYNIC was chosen as the proper BFCA which attached to the N-terminal of the peptide at position 0 in the sequence without adverse effects on its receptor affinity. The use of HYNIC permits a good labeling efficiency and stability beside the possibility to control the lipophilicity and pharmacokinetics of the labeled compound by using various coligands as explained by other researchers (50-52). Among the various available coligands, it has been confirmed that the use of both EDDA and Tricine together results in the optimized stability and labeling yield in comparison to each of them alone or other coligands (27, 53-55). Therefore, we chose the exchange labelling approach, with both tricine and EDDA present together. In our study, a good labeling yield (>95%) without any remarkable impurities was achieved which is consistent with reports by others (31, 56-58). Also, the results of this study are correlated with the mentioned facts that the EDDA-Tricine coligand strategy showed good stability and low percent of protein binding (29, 55, 58).

*In-vitro* cell line evaluations indicated an acceptable cell uptake in U373MG cells after 0.5 h which increased during 4 h and the ratio of specific uptake to total uptake was about 60%. An interesting observation from internalization study was the saturation of specific surface-bound activity after 1 h while the ratio of specific internalized activity to specific surface-bound activity was increased during the time (Figure 4a, 4b). This finding could be a result of the receptor mediated endocytosis (RME) mechanism which also called clathrin-dependent endocytosis (CME). As known, binding of SP to NK_1_R leads to internalization by CME mechanism to the acidified endosomes where the complex dissociates. Consequently, SP is degraded and NK_1_R is re-expressed on the cell surface (59). CME could be the responsible mechanism for internalization of ^99m^Tc labeled HYNIC-Tyr^8^-Met(O)^11^-SP on U373MG cells in the same way. The internalization ability of a radioligand is important to make it the ideal as it can no longer take part in the equilibrium process and also it guarantees intracellular delivery of the radioisotope (60). Saturation binding experiment confirmed the receptor specific binding of ^99m^Tc labeled HYNIC-Tyr^8^-Met(O)^11^-SP to the surface of U373MG cells.

In the biodistribution study high kidney uptake of the radiolabeled peptide could be a consequence of high hydrophilicity of the EDDA-^99m^Tc-HYNIC-peptide. However, the lysosomal trapping of the metabolized radiopeptide may be another reason for high uptake and prolonged retention of activity in kidneys(61). The moderate clearance of the radiolabeled peptide from the circulation could be caused by its 44% protein binding. Although the uptake in liver was lower than kidneys, data from uptake changes during the time shows a small portion of the hepatobiliary pathway in addition to renal excretion. Decreased percent (D%) inuptakes of tissues during the time demonstrates these findings. It could be explained that the activity has been washed out from the organs with higher D% to the organs with lower D% throughout the elimination process. Also it could explain the pattern of activity in the large intestine due to final excretion of radiometabolites and transit through the GI tract.

It is notable that the mucosal tissues are approximately normal targets of the mentioned radiopeptide. So, maybe there was a relation between mucosal excretion potency of organs and their ability to absorb the EDDA-^99m^Tc-HYNIC-Tyr^8^-Met(O)^11^-SP. This claim could be verified by reported NK_1_R expression sites (15, 62, 63). The moderate activity of stomach and salivary glands in comparison to other organs might be due to presence of some degree of free ^99m^TcO_4_^-^ but expression of NK_1_R in the mucosa of these organs is another possibility. However in blocking study non significant decreased of activity was observed in them. In blocked tumor and intestines the reduction was significant which indicate specific targeting in these organs.

As it is obvious the peptide backbone of mentioned radioconjugate makes it more hydrophilic and larger than the ideal to cross the Blood Brain Barrier(BBB) via intravenous (IV) injection. On the other hand, the Glioblastomas are CNS tumors. Despite the damage to BBB caused by tumor invasion, lack of penetration through the intact BBB is a disadvantage of this new radiopeptide. However, this radiolabeled compound could also be injected intrathecally for follow up of the recurrence of GBM tumors after surgery or radiotherapy, instead of invasive tissue sampling methods. Also, this radiolabeled compound could be used by IV injection for diagnosis of NK_1_R positive metastases and other tumors namely breast carcinoma, colorectal carcinoma, pancreatic carcinoma, retinoblastoma, neuroblastoma, melanoma, laryngeal carcinoma, and oral squamous cell carcinoma. Additionally there are some specific and non-destructive pathways by receptors or carriers for many peptides and proteins to penetrate the BBB. This process has not yet been tested for SP (64). This new radiolabeled compound has the potential to use for detection of primary recurrences or metastases of the cancers expressing NK_1_Rs.

## Conclusion

In this study, we described the synthesis and radiolabeling of anew HYNIC containing analogue of SP with a high receptor specific affinity for detection of NK_1_R expressing tumors. Furthermore, a convenient method with high yields of synthesis and labeling was presented. Further investigations are needed to confirm the potential advantage of this new radiolabeled compound in well designed *in-vivo* experiments and clinical trials.
